# Comparative Evaluation of Allometric, Machine Learning, and Ensemble Approaches for Modeling Dynamic Structure–Fresh Weight Relationships in Sweet Pepper

**DOI:** 10.3390/plants15071063

**Published:** 2026-03-31

**Authors:** Jun Hyeun Kang, Taewon Moon

**Affiliations:** Smart Farm Research Center, Korea Institute of Science and Technology, Gangneung 25451, Republic of Korea; mallejh@kist.re.kr

**Keywords:** allometric model, data augmentation, ensemble learning, fresh weight estimation, machine learning, sweet pepper

## Abstract

Accurate fresh weight (FW) estimation is essential for growth monitoring and yield prediction in greenhouse fruit vegetables, but remains challenging due to the dynamic allocation between vegetative and reproductive organs. This study aimed to systematically evaluate modeling strategies for FW estimation in sweet pepper and identify which approach is most suitable under conditions of dynamic biomass partitioning. Non-destructive morphological measurements were collected under greenhouse cultivation, and allometric models based on geometric equations were established as baselines. Their performance was compared with machine learning (ML) models and ensemble learning frameworks. To address limited data availability, numerical data augmentation with Gaussian noise and a variational autoencoder was applied. Among the allometric models, the stick model combined with a sigmoid function showed the highest performance, with an $R^{2}$ of 0.80 for shoot FW and 0.54 for fruit FW. All ML models outperformed the allometric models, and the ensemble model achieved the highest predictive accuracy, with an $R^{2}$ of 0.96 for shoot FW and 0.89 for fruit FW. Data augmentation further improved predictive performance across all ML models, particularly for fruit FW prediction. Feature contribution analysis revealed that temporal progression was the dominant predictor of fruit FW, while structural traits played the primary role in shoot FW estimation. Ensemble-based ML, combined with data augmentation, provides a methodological framework for non-destructive FW estimation of sweet pepper in controlled environments such as greenhouses and smart farming systems.

## 1. Introduction

Since plant fresh weight (FW) is a fundamental indicator of plant growth and development, FW estimation has been studied for growth monitoring, yield prediction, and resource management in horticultural systems [1,2,3,4]. In fruit vegetables, FW variation is characterized by repeated harvesting and continuous redistribution of assimilates between vegetative and reproductive organs. Shoot growth continues alongside fruit production, with the relative contribution of vegetative and reproductive organs to total FW dynamically shifting over time [5,6]. These dynamics complicate a straightforward interpretation of the relationship between morphological traits (e.g., plant height, stem diameter, and stem length) and FW. Although destructive measurement can accurately quantify FW at a specific time point, it is not suitable for tracking the same plants and collecting commercial cultivation data. Consequently, practical growth monitoring depends on non-destructive morphological measurements [7,8]. However, converting these measurements into a reliable FW estimation remains challenging, as dynamic biomass allocation between vegetative and reproductive organs obscures the relationship between morphological traits and FW.

Similar challenges in converting non-destructive traits have been addressed in forestry through allometric equations [9,10,11]. These equations estimate FW from external structural traits by expressing trait–FW relationships as mathematical scaling functions. Allometric modeling has provided a biologically interpretable framework for linking plant structure and productivity; nevertheless, allometric relationships are typically formulated as fixed mathematical functional forms [10], which may not be applicable to fruit vegetables. In these crops, developmental transitions and reproductive shifts often lead to time-dependent scaling exponents and proportional relationships [5,12]. This suggests that static allometric equations should be modified for FW estimation in fruit vegetables with dynamic partitioning.

Since machine learning (ML) can approximate complex nonlinear relationships without predefined structural assumptions, it could provide a flexible framework for modeling dynamically changing structure–FW relationships [13]. Previous studies on plant FW estimation have focused on remote sensing or image-derived features [14,15]. Systematic evaluation of ML approaches using non-destructive morphological measurements in greenhouse fruit vegetables remains limited. Therefore, a comparison between these interpretable allometric models and flexible ML approaches could clarify their respective advantages for FW estimation.

One of the challenges in ML approaches is the substantial time investment required for model selection and hyperparameter tuning to achieve the desired level of accuracy for a given task [16,17]. ML models can capture complex relationships, but their predictions may become sensitive to data preprocessing and model parameterization, particularly when training data is limited [18,19]. Ensemble learning addresses this issue by integrating multiple predictive models to reduce variance and improve robustness [14,20,21]. This suggests that ensemble strategies may provide a useful framework for stabilizing FW prediction in systems with dynamic structure–FW relationships.

In addition, training ML models requires sufficiently representative datasets. However, destructive FW sampling in agricultural research is labor-intensive and temporally constrained, often resulting in limited sample sizes [22]. Under such small-sample conditions, flexible models may capture dataset-specific patterns rather than generalizable structure–FW relationships [22,23]. Data augmentation strategies provide one potential solution to expand effective training distribution without additional destructive sampling [24].

In this study, we aimed to systematically evaluate which modeling approaches are most suitable for FW estimation under dynamic biomass allocation conditions in fruit vegetables, using non-destructive morphological measurements collected under greenhouse conditions. Allometric models were first established as interpretable baselines. Their performance was then compared with ML models of increasing structural flexibility. Particular emphasis was placed on ensemble learning frameworks to assess whether integrating multiple predictive models improves accuracy and predictive stability relative to single-model approaches. Data augmentation using a variational autoencoder was incorporated to mitigate small-sample limitations. Through structured comparison of modeling paradigms, this study provides biologically interpretable insights into FW estimation by elucidating the relative roles of temporal and structural factors under dynamic biomass partitioning in fruit vegetables.

## 2. Results

### 2.1. Distribution Similarity Between Original and Augmented Data

**Figure 1:**
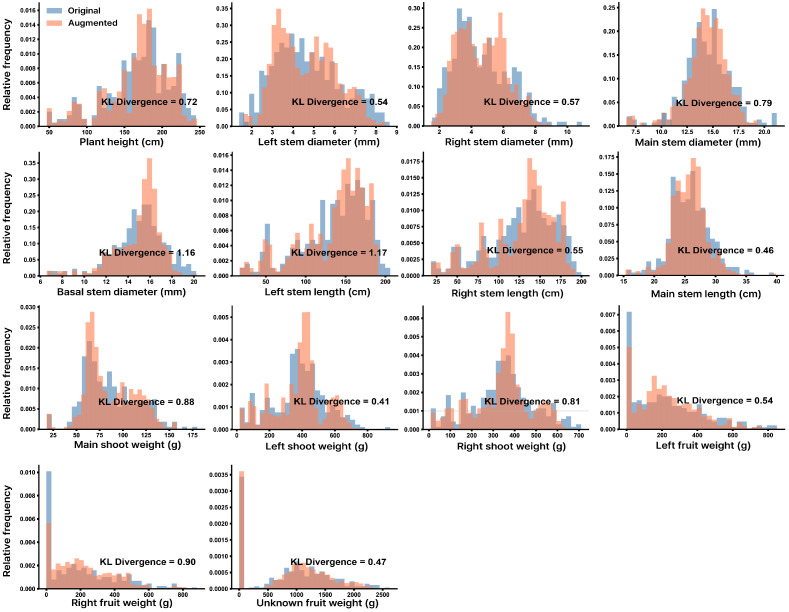
Histogram of original and augmented data using Gaussian noise and variational autoencoder.

Three modeling approaches for FW estimation were compared: allometric models, ML models, and ensemble model. Data augmentation was applied to the training data to address limited sample sizes. To evaluate whether the augmented data preserved the statistical characteristics of the original dataset, the distributions of morphological traits and FW variables were compared between original and augmented data (Figure 1). Histograms showed that the augmented samples closely followed the distribution patterns of the original data across most variables. Across all variables, the average KL divergence was 0.71.

### 2.2. Allometric Model Performance

**Figure 2:**
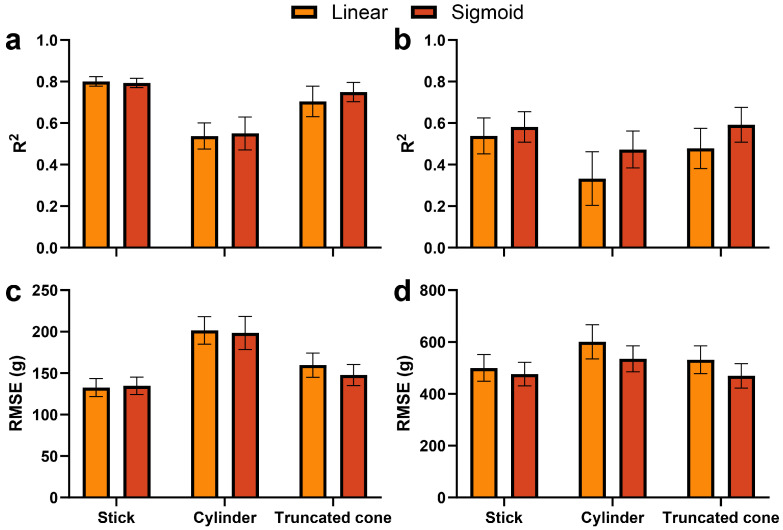
Performance of allometric models using stick, cylinder, and truncated cone geometries combined with linear and sigmoid functions. (**a**,**b**) $R^{2}$ for shoot and fruit fresh weight (FW); (**c**,**d**) RMSE for shoot and fruit FW. Bars represent standard deviation across repeated cross-validations.

Plant volume was approximated using three geometric representations: stick (height only), cylinder, and truncated cone. Each was combined with linear or sigmoid scaling functions (see Section 4.3.1). The predictive performance of allometric models varied depending on the geometric representation (Figure 2). For shoot FW, the stick model showed the highest accuracy among the three geometric representations, with $R^{2}$ close to 0.8 for both linear and sigmoid functions (Figure 2a). The cylinder model showed lower $R^{2}$, approximately 0.5, while the truncated cone model showed intermediate performance. A similar pattern was observed for fruit FW (Figure 2b). The stick model achieved higher $R^{2}$ than the cylinder model, whereas the truncated cone model showed intermediate performance.

The RMSE also showed a tendency similar to that of the $R^{2}$ results (Figure 2c,d). For shoot FW, the stick model produced the lowest RMSE, followed by the truncated cone model, while the cylinder model showed the largest errors. For fruit FW, the truncated cone model produced lower RMSE than the stick model, whereas the cylinder model showed the largest prediction errors. Across the three allometric models, the sigmoid function produced slightly higher $R^{2}$ than the linear model in several cases, particularly for fruit FW prediction.

### 2.3. Machine Learning Model Performance

**Figure 3:**
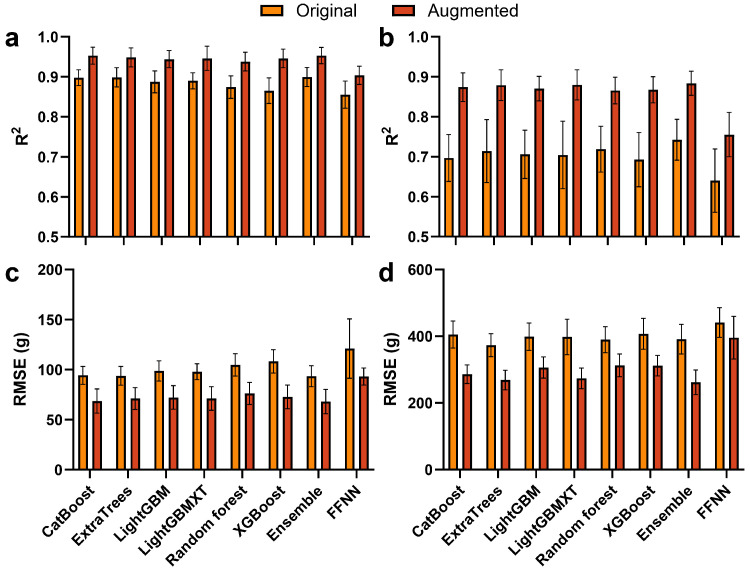
Performance comparison of machine learning models trained with original and augmented datasets. (**a**,**b**) $R^{2}$ for shoot and fruit fresh weight (FW); (**c**,**d**) RMSE for shoot and fruit FW. Bars represent standard deviation across repeated cross-validations.

Multiple ML models, including tree-based methods (e.g., CatBoost, LightGBM, XGBoost, Random Forest), a feed-forward neural network, and a stacked ensemble, were trained for shoot and fruit FW prediction (see Section 4.3.2). Their predictive performance was compared between models trained with the original and augmented datasets (Figure 3). For shoot FW prediction, models trained with the augmented dataset showed higher $R^{2}$ than those trained with the original dataset across all algorithms (Figure 3a). The $R^{2}$ of models trained with the original dataset ranged from approximately 0.86 to 0.90, whereas models trained with the augmented dataset produced values above 0.92 for most models.

For fruit FW prediction, the augmented dataset also produced higher $R^{2}$ across all models (Figure 3b). The $R^{2}$ of models trained with the original dataset ranged from approximately 0.64 to 0.75, whereas models trained with the augmented dataset ranged from approximately 0.81 to 0.89. RMSE showed corresponding differences between the two datasets. For shoot FW prediction, models trained with the augmented dataset produced lower RMSE values than those trained with the original dataset across all models (Figure 3c). A similar pattern was observed for fruit FW prediction, where RMSE values were lower for models trained with the augmented dataset (Figure 3d).

### 2.4. Comparison Between Allometric and Machine Learning Models

**Figure 4:**
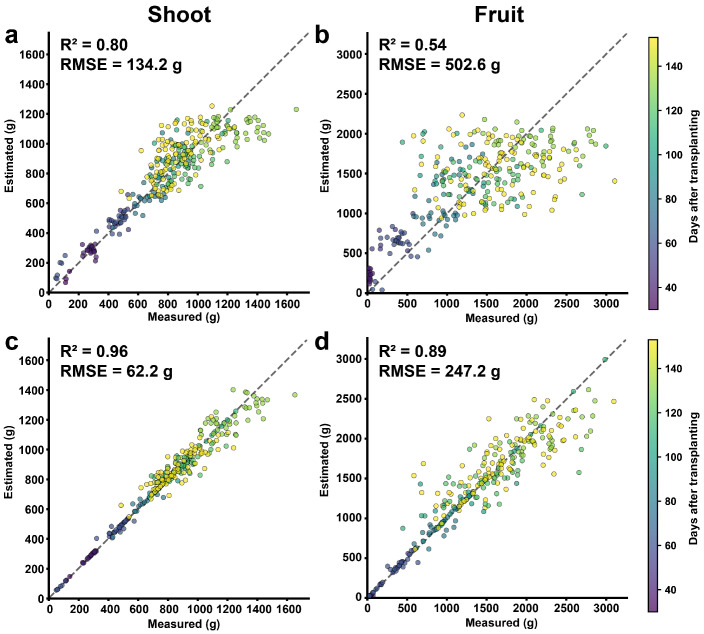
Comparison of fresh weight (FW) predictability using the best-performing allometric (stick) and ensemble learning models. (**a**,**b**) Shoot and fruit FW predicted by the stick allometric model; (**c**,**d**) shoot and fruit FW predicted by the ensemble model. Point color indicates days after transplanting. The dotted line represents the 1:1 line between measured and estimated values.

The prediction results of the best-performing allometric model and the best-performing machine learning model were compared using scatter plots of measured and estimated values (Figure 4). The stick model with sigmoid function showed the highest performance among the tested allometric models. The $R^{2}$ was 0.80 and RMSE was 134.2 g for shoot FW (Figure 4a). For fruit FW, the $R^{2}$ was 0.54 and RMSE was 502.6 g (Figure 4b). For the ML, the ensemble model showed the highest predictive performance among the evaluated MLs. The $R^{2}$ was 0.96 and RMSE was 62.2 g for shoot FW (Figure 4c). For fruit FW, the $R^{2}$ was 0.89 and the RMSE was 247.2 g (Figure 4d), representing an approximately 65% increase in $R^{2}$ compared with the allometric model.

### 2.5. Feature Contribution and Model Interpretation

**Figure 5:**
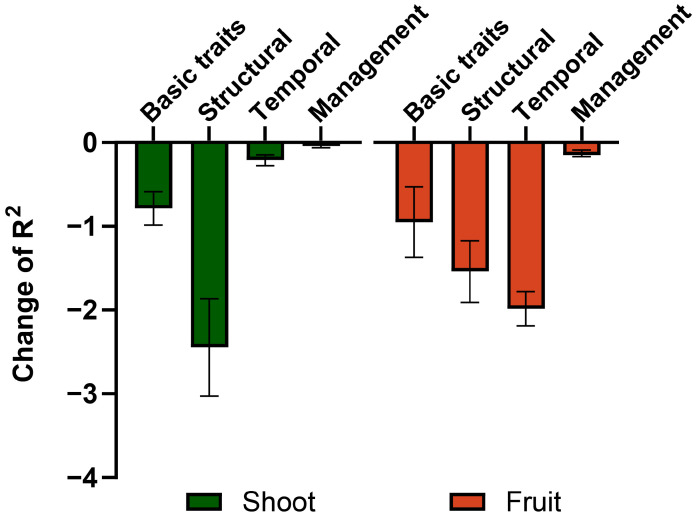
Change in $R^{2}$ after excluding each feature group from the ensemble model for estimating shoot and fruit fresh weight of sweet pepper. The $R^{2}$ of full model was 0.96 (shoot) and 0.89 (fruit).

To quantify the contribution of different feature groups to FW prediction, an ablation analysis was conducted using the ensemble model (Figure 5). The decrease in $R^{2}$ was evaluated after removing each feature group from the full model. For shoot FW prediction, removal of structural variables resulted in the largest decrease in $R^{2}$, indicating that plant structural traits play a dominant role in predicting vegetative FW. Basic traits showed the second largest contribution, whereas temporal variables had a relatively smaller effect, and management variables had minimal impact on model performance.

For fruit FW prediction, removal of temporal variables produced the largest decrease in $R^{2}$, confirming that developmental stage is the primary driver of fruit FW accumulation. Structural variables showed the second largest contribution, followed by basic traits, while management variables contributed the least to prediction accuracy.

**Figure 6:**
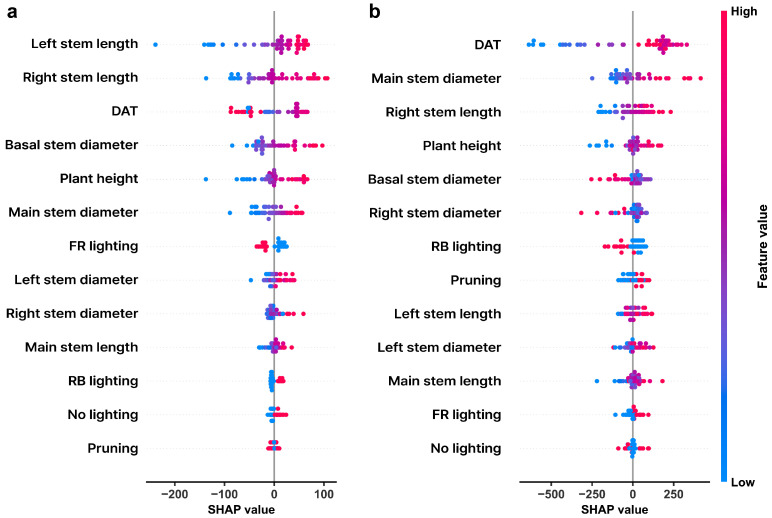
SHAP summary plots showing feature contributions in the ensemble model. (**a**) Shoot fresh weight prediction. (**b**) Fruit fresh weight prediction. Feature colors represent the magnitude of the feature value.

To further examine the contribution of individual variables, SHAP (Shapley Additive Explanations) analysis was conducted using the trained ensemble models, which was the best-performing model, to quantify individual feature contributions (Figure 6). For shoot FW prediction, structural factors showed the largest SHAP values, with stem length variables (left and right stem length) ranking as the most influential features. Basal stem diameter, plant height, and main stem diameter also contributed substantially, while DAT showed a moderate contribution compared with structural factors. Management-related factors had low impact on prediction.

For fruit FW prediction, DAT exhibited the largest SHAP magnitude among all variables, indicating that developmental stage is the dominant factor influencing fruit FW. Structural factors, including main stem diameter, stem length, and plant height, also showed notable contributions but were secondary to temporal variables. Management-related variables such as lighting treatments and pruning consistently showed low SHAP contributions.

## 3. Discussion

### 3.1. Limitations of Allometry in Crops with Dynamic Fresh Weight Partitioning

Allometric models provided interpretable baselines, but their predictive accuracy was limited for sweet pepper (Figure 4). The best-performing configuration was the stick model combined with a sigmoid growth function, which showed moderate performance for shoot FW estimation ($R^{2}=0.80$), whereas performance for fruit FW was substantially lower ($R^{2}=0.54$). Interestingly, the stick model, which considers only stem length, outperformed more detailed allometric models. This contrasts with previous study across various tree species, where diameter-based allometric models achieved higher accuracy than stick models [9,10]. Such differences likely reflect the growth architecture of sweet pepper, in which canopy development progresses mainly along the vertical axis; therefore, plant height is closely associated with FW accumulation. More fundamentally, the limitation arises not from the allometric concept itself but from applying static equations to a temporally dynamic system. In sweet peppers, FW allocation between vegetative and reproductive organs changes substantially due to fruit set, rapid fruit expansion, and repeated harvest events governed by source–sink interactions and assimilate competition [6,12]. Consequently, although allometric models remain useful for rapid shoot-level approximation, their predictive capability is limited in estimating fruit weight. This was especially evident in the high-mass range, where the model underestimated fruit FW (Figure 4b), reflecting the difficulty of static scaling relationships in capturing rapid fruit expansion.

### 3.2. Data Augmentation Improved Predictive Accuracy Under Limited Agricultural Data

The results of this study showed that data augmentation improved model accuracy under limited destructive sampling (Figure 3). Across all evaluated models, models trained with the augmented dataset achieved higher $R^{2}$ and lower RMSE than those trained with the original dataset. The improvement was especially pronounced for fruit FW prediction, where $R^{2}$ increased from approximately 0.63–0.74 to 0.78–0.89. This larger gain is biologically meaningful because fruit FW is strongly influenced by dynamic source–sink transitions and repeated fruit harvest, which are difficult to represent with sparse observations alone.

Agricultural datasets are often limited in size because destructive FW measurements are labor-intensive and cannot be repeatedly performed on the same plants. Under such conditions, machine learning models may easily overfit to the limited training samples [25]. VAEs provide a practical solution to this problem by learning the probability distribution of the training data and generating new synthetic observations that follow the same latent distribution [26].

In the present study, the augmented samples reproduced the statistical characteristics of the original dataset (Figure 1). Distributional comparisons indicated that the augmented data captured the major patterns of the original observations (KL divergence ≤1), suggesting that the generative model preserved the overall feature distribution while introducing moderate variability [27]. Consistent with previous studies, the generated samples remained close to the original data space without introducing obvious unrealistic values [24,28].

However, matching marginal distributions alone does not guarantee that multivariate relationships between variables are fully preserved [29,30]. Synthetic data may introduce unrealistic combinations of traits or distort underlying biological relationships, which could affect model interpretability [31]. In this study, although no obvious unrealistic patterns were observed, the preservation of biologically meaningful relationships between variables, such as coordination among morphological traits or source–sink dynamics, was not explicitly validated. Therefore, further validation approaches, including joint distribution analysis or incorporation of physiological constraints, would be necessary to ensure the biological consistency of augmented data. Despite these limitations, the augmented dataset improved predictive accuracy and reduced overfitting, indicating that data augmentation can be a useful approach for FW prediction in data-limited agricultural systems when applied with appropriate caution.

### 3.3. Ensemble Learning Provided the Highest Predictive Accuracy Across Fresh Weight Targets

All ML models outperformed the traditional allometric models in predicting both shoot and fruit fresh weight (Figure 2 and Figure 3). While allometric approaches offer interpretable geometric approximations of plant structure, they rely on predefined functional forms that may not fully capture the dynamic allocation of FW during fruit development. Such assumptions were insufficient for sweet pepper, where FW accumulation is influenced by dynamic developmental processes. However, ML models could integrate heterogeneous features and thus capture nonlinear relationships that govern FW accumulation.

Among the evaluated models, the ensemble model achieved the highest accuracy. Ensemble learning aggregates predictions from multiple base learners and is known to reduce variance and improve robustness by combining complementary model structures. In the present study, this approach substantially improved fruit FW estimation, increasing $R^{2}$ from 0.54 to 0.89 relative to the allometric model. The improvement was particularly evident for high fruit FW values, where the allometric model underestimated FW (Figure 4b). By contrast, the ensemble model successfully captured the nonlinear and temporally dynamic relationships associated with fruit development (Figure 4d). The deep learning model (FFNN), which is widely known to achieve strong predictive performance across many domains [32,33], did not outperform the ensemble model in this study. Deep learning methods typically show advantages in large-scale and high-dimensional datasets, whereas their benefits are less consistent for relatively simple tabular datasets such as those used in this study [18,22].

To further disentangle the contributions of model structure and data augmentation, additional comparisons were performed. When trained on the original dataset, the ensemble model achieved an $R^{2}$ of approximately 0.90 for shoot FW and 0.74 for fruit FW, compared with 0.80 and 0.54 for the best-performing allometric model, respectively. This indicates that the change in model structure contributed to an increase of approximately 0.10 for shoot FW and 0.20 for fruit FW.

Subsequent application of data augmentation further improved model performance, increasing $R^{2}$ from approximately 0.90 to above 0.96 for shoot FW and from 0.74 to approximately 0.89 for fruit FW. These results indicate that both model flexibility and data augmentation contributed to performance improvement, with model structure providing the primary gain and data augmentation offering additional improvements.

### 3.4. Organ-Specific Contributions of Structural and Temporal Factors in Fresh Weight Prediction

Feature contribution analysis provided insight into the relative importance of different variable groups in FW prediction (Figure 5 and Figure 6). The ablation analysis revealed that structural variables were the most influential predictors for shoot FW, whereas temporal variables were the most influential predictors for fruit FW. This indicates that shoot FW is more strongly associated with plant structural development, while fruit FW accumulation is primarily governed by developmental progression. However, this does not imply that FW accumulation is determined solely by time, as other non-temporal variables also contributed substantially to model performance.

For shoot FW prediction, structural variables such as stem length and stem diameter showed the largest contribution, followed by basic traits. This suggests that shoot FW can be effectively predicted using morphological variables.

For fruit FW prediction, temporal factor remained the most influential predictor, whereas structural factors showed the second largest contribution, followed by basic traits, while management factors had relatively limited impact on model performance (Figure 5). This suggests that cumulative fruit FW is primarily governed by developmental progression and plant structural status, rather than being directly influenced by management conditions within the current dataset. Supplemental lighting treatments, particularly red–blue LED interlighting (RB) and red–blue interlighting supplemented with far-red light (FR), have been reported to influence assimilate allocation and fruit sink strength, thereby affecting yield [34,35]. However, in the present study, the contribution of management factors to fruit FW prediction was relatively limited. One possible explanation is that cumulative fruit FW inherently integrates growth over time, which may obscure the direct effects of management treatments on individual fruit development. As a result, potential variations in sink strength induced by lighting conditions may not be explicitly captured in the current dataset. Future studies incorporating fruit-level measurements, such as individual fruit weight, may provide more detailed insights into how lighting treatments influence sink strength and yield formation.

In contrast, SHAP analysis mainly highlighted temporal and morphological factors at the individual-feature level (Figure 6). This difference arises from the methodological characteristics of the two approaches. Ablation analysis evaluates the change in model performance after removing a feature group, thus reflecting the global contribution of factors, including their interactions with other predictors [36]. SHAP values quantify the marginal contribution of individual factors within the trained model [37]. The SHAP analysis further provides insight into the role of individual variables in FW prediction. For shoot FW, stem lengths showed the highest SHAP values, indicating that vegetative biomass is primarily determined by plant structural characteristics. This supports the ablation results, which identified structural variables as the dominant contributors to shoot FW prediction (Figure 5). For fruit FW, DAT exhibited the largest SHAP magnitude among all variables, confirming that developmental stage is the primary driver of cumulative fruit FW. Structural variables also showed substantial contributions but were secondary to temporal variables, suggesting that fruit biomass accumulation is strongly linked to time-dependent growth processes.

The applicability of the proposed framework may be influenced by specific cultivation conditions and plant architecture. In this study, the model was developed using sweet pepper plants trained to a two-stem system under greenhouse conditions. However, alternative training systems, such as three-stem cultivation, change plant architecture, light interception, and underlying physiological processes, potentially affecting model performance [38,39]. In addition, although multiple cultivars were included, further expansion to a wider range of genotypes may improve model generalizability. Moreover, key physiological processes specific to fruit and vegetable production, such as flower abortion, which can substantially influence yield formation, were not explicitly considered in the current model. Incorporating such processes, together with more detailed organ-level measurements, may further enhance the robustness and biological relevance of FW prediction in future studies.

## 4. Materials and Methods

### 4.1. Experimental Design and Plant Material

The experiment was conducted with sweet pepper (*Capsicum annuum* var. *annuum*) grown in a Venlo-type greenhouse at the experimental farm of Seoul National University (Suwon, Republic of Korea; 37.3° N, 127.0° E). The experiment was conducted for five growing seasons with three commercial cultivars (Table 1). Various cultivars were included to improve the generalization of ML models.

The seedlings were transplanted into rockwool slabs (Grodan GT Master, Grodan, Roermond, The Netherlands) and trained to two main stems using vertical strings. Plants were fertigated using an open-loop nutrient system with PBG solution (electrical conductivity 2.6–3.0 dS m^−1^; pH 5.5–6.5). Irrigation (133 mL per plant) was triggered when cumulative solar radiation reached 50 J cm^−2^.

Three lighting treatments (no interlighting (CT), red–blue LED interlighting (RB), and red–blue interlighting supplemented with far-red light (FR)) were intended to induce various growth conditions to train ML models with improved generalization capability. To prevent light contamination, the greenhouse was partitioned into separate zones using shading curtains. Interlighting modules were installed along the growing beds at 90 and 110 cm above the substrate. The red-to-blue photon ratio was fixed at 8:2, delivering 100 μmol m^−2^ s^−1^ measured at 20 cm from the modules. In the FR treatment, additional far-red light was supplied at 60 μmol m^−2^ s^−1^. Supplemental lighting was initiated once shoot meristems reached approximately 80 cm and was applied daily for 12 h (06:00–18:00).

### 4.2. Data Collection and Target Definition

**Figure 7:**
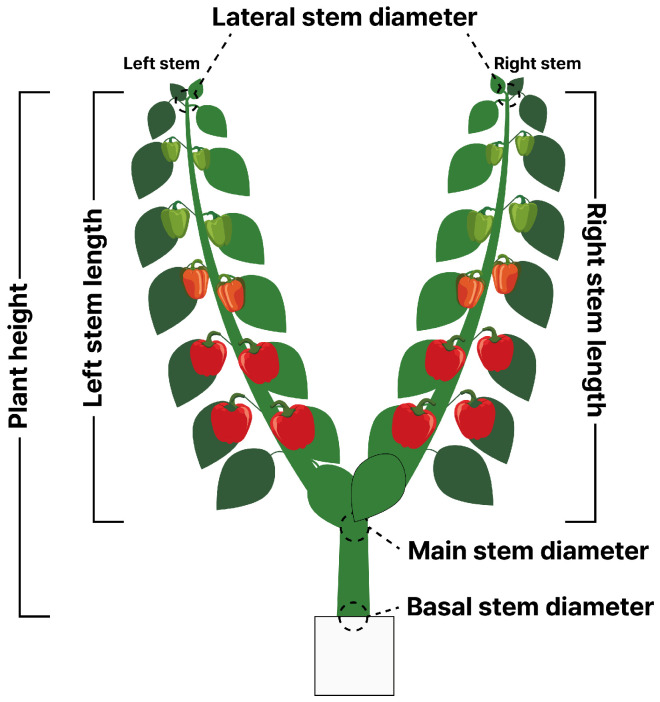
Collected morphological traits and variables.

Destructive sampling was conducted at predefined intervals throughout the cultivation period (Table 1). At each sampling event, morphological and architectural traits were recorded prior to FW measurement. The recorded variables included plant height; stem diameters measured at upper, middle, and lower positions of the main stem; and segment lengths representing left, right, and middle stems (Figure 7). Days after transplanting (DATs) were included as a temporal factor representing developmental stage.

Following measurement of morphological traits, plants were separated into vegetative and reproductive organs, and the fresh weight was recorded for each organ. Shoot fresh weight (shoot FW) was defined as the fresh weight of vegetative plant tissues excluding harvested fruits. Fruit fresh weight (fruit FW) was defined as the cumulative fresh weight of harvested fully ripened fruits up to the sampling date. These two variables were used as regression targets in subsequent modeling analyses.

### 4.3. Modeling Framework Overview

**Figure 8:**
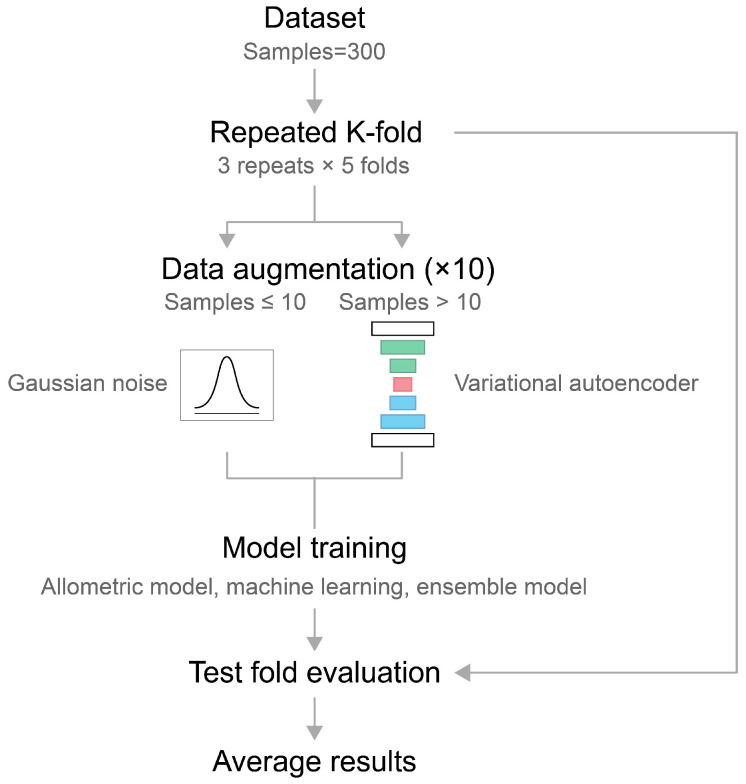
The modeling workflow used for fresh weight estimation. In the variational autoencoder, green, red, and blue boxes indicate the encoder, latent space, and decoder, respectively.

To systematically evaluate modeling strategies for FW estimation, three categories of models were examined within a unified analytical framework: allometric modeling, ML modeling, and ensemble learning (Figure 8). Allometric models were constructed as structurally interpretable baselines, representing FW as explicit functions of geometric approximation derived from morphological traits. ML models were implemented to flexibly approximate relationships between the morphological traits and shoot and fruit FWs without predefined structural assumptions. The ensemble model was applied to integrate multiple ML models to reduce variance and improve predictive stability. All modeling approaches were trained and evaluated under an identical cross-validation scheme, enabling direct comparison of predictive performance and robustness across modeling methods.

#### 4.3.1. Allometric Modeling

**Figure 9:**
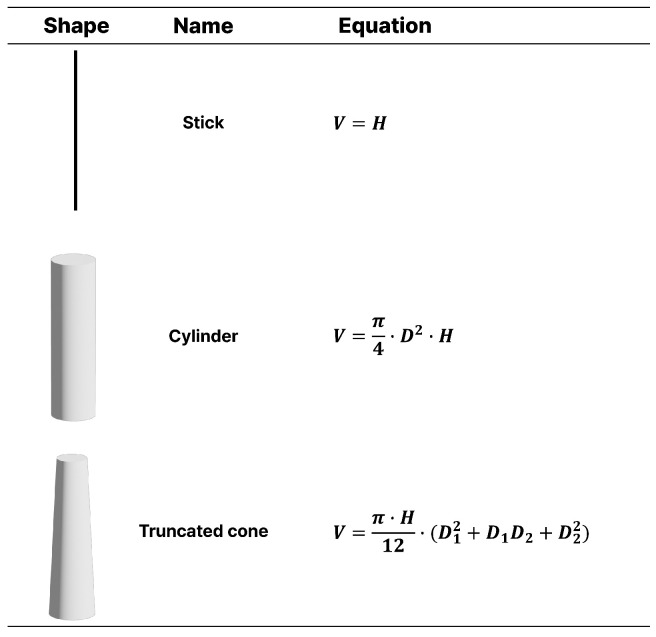
Geometric representations used for allometric modeling.

To establish interpretable structural baselines, plant volume was approximated using three geometric representations: stick, cylinder, and truncated cone (Figure 9), which were selected to approximate the structural architecture of sweet pepper plants [10]. Since sweet pepper plants are usually grown in multiple stems, the plant was approximated using three longitudinal segments of left, right, and middle (Figure 7). Each stem was simply summed to obtain a whole-plant volume (*V*).

Using each volumetric proxy, the shoot and fruit FWs were modeled independently using two alternative functional forms. First, a linear model was applied to represent proportional scaling between volume and FW:

(1) FW=αV+β

where α and β are fitted parameters. To account for potential nonlinear saturation during plant development, a logistic sigmoid function was also evaluated:

(2) $$\mathrm{FW}=\frac{L}{1+\exp\left[-k(V-V_{0})\right]}$$

where *L* represents the asymptotic maximum FW, *k* denotes the growth rate parameter, and $V_{0}$ is the inflection point of the growth curve. This equation allows the allometric relationship to reflect developmental dynamics in FW accumulation, particularly under conditions where growth approaches saturation.

The model parameters were estimated using least-squares optimization. When convergence of the sigmoid model was unstable due to the limited sample size or poor initialization, the linear model was adopted to ensure stable baseline estimation. These allometric models served as structurally interpretable reference models against which data-driven approaches were compared.

#### 4.3.2. ML Approach and Ensemble Learning

a. Feature construction

The input factors were organized into four feature categories according to their biological and practical functions in the monitoring of the status of the crop (Table 2). Basic traits represent fundamental morphological parameters that are routinely measured during non-destructive measurement. Structural traits describe more detailed characteristics of the plant, which generally require additional measurements beyond Basic traits. DATs were used as a temporal factor for crop developmental stage. Management factors represent cultivation practices such as pruning.

b. Model training and ensemble model construction

ML models were implemented using the AutoGluon framework (AutoGluon v1.5.0; Amazon Web Services, Seattle, WA, USA; [40]). Each regression model was trained for shoot FW and fruit FW. AutoGluon automatically trained multiple base models including tree-based methods, linear models, and neural network models. Based on cross-validated performance, a stacked weighted ensemble model was constructed to combine predictions from individual base models.

The ensemble weights were optimized to minimize prediction error on validation folds, thereby reducing variance and improving predictive robustness. The final ensemble model consisted of four base learners: LightGBMXT, RandomForestMSE, CatBoost, and NeuralNetTorch. Among these, LightGBMXT and CatBoost contributed the largest weights (0.3913 and 0.3043, respectively), followed by NeuralNetTorch (0.2609), while RandomForestMSE contributed a smaller weight (0.0435). This weighting scheme reflects the relative predictive performance of each model during the stacking process.

In addition, a feed-forward neural network (FFNN) was implemented to evaluate deep learning-based regression performance. The FFNN consisted of two hidden layers with 32 and 8 neurons, respectively. Each hidden layer was followed by batch normalization, ReLU activation, and dropout regularization. The output layer consisted of a single neuron for regression. The network was trained using an Adam optimizer with a mean squared error loss function. The maximum number of training epochs was set to 500, with early stopping applied when validation loss did not improve for 80 consecutive epochs.

#### 4.3.3. Data Augmentation

Since the number of destructive FW samples was limited, numerical data augmentation was applied to expand the valid training data. Augmentation was performed independently within each season, treatment, and DAT group to preserve developmental stage distributions and treatment-specific growth patterns.

Continuous variables describing plant morphology were used for the generative process, while categorical and discrete variables were excluded from model training and reassigned after sample generation. Continuous features were standardized prior to model training. In this study, Gaussian noise and a variational autoencoder (VAE) were utilized for data augmentation (Table 3).

The threshold for selecting the augmentation method was determined empirically. Specifically, multiple candidate thresholds (*n* = 3, 5, 7, and 10) were evaluated by comparing the statistical similarity between original and augmented datasets using KL divergence. A threshold of *n* = 10 resulted in the lowest KL divergence, indicating the best preservation of the original data distribution. Therefore, VAE-based augmentation was applied only when the number of samples exceeded 10, while Gaussian noise was used for smaller sample sizes.

a. Gaussian noise approach for small samples (n≤10)

Gaussian noise was applied to groups with ten or fewer observations to preserve local statistical characteristics of the original data distribution [41]. Augmented data were generated by randomly selecting original observations and perturbing their continuous features. with Gaussian noise scaled by the feature-wise standard deviation. This approach preserves the local structure of the data distribution while introducing small stochastic variations. Categorical and discrete variables, such as DATs and pruning status, were reassigned according to the group to which the augmented samples belonged.

b. VAE approach for large samples (n>10)

A VAE was used for groups containing more than ten observations to generate synthetic samples by learning the underlying distribution of the original data and sampling from the latent representation [24,26,42]. The VAE consisted of fully connected encoder and decoder networks with two hidden layers each. Batch normalization, LeakyReLU activation, and dropout regularization were applied to improve training stability and prevent overfitting.

The encoder mapped the input features into a latent representation characterized by a latent mean (μ) and log-variance ($\log\sigma^{2}$). A reparameterization trick was used to enable backpropagation through stochastic sampling [26]. The decoder reconstructed feature vectors from the latent space.

Model training minimized a combined loss consisting of reconstruction error and Kullback–Leibler (KL) divergence [26,27]. After training, synthetic samples were generated by sampling latent vectors from a standard normal distribution and passing them through the decoder network. For each group, synthetic samples were generated up to ten times the size of the original observations. To prevent information leakage, data augmentation was applied only to the training folds within the cross-validation procedure.

c. Evaluation metric for similarity of augmented data

To evaluate the similarity between the distributions of the original and augmented datasets, the KL divergence was calculated [27]. KL divergence measures the difference between two probability distributions and was used to quantify how closely the augmented data followed the distribution of the original dataset.

### 4.4. Cross-Validation and Evaluation Metrics

#### 4.4.1. Cross-Validation Strategy

Model performance was evaluated using repeated *K*-fold cross-validation (5 folds × 3 repeats). In each fold, only original observations were used to define training and test splits. Test folds consisted exclusively of original data to ensure unbiased performance estimation. Augmented samples were added only to training folds and were excluded if corresponding to test-fold groups.

Out-of-fold (OOF) predictions were obtained during the repeated *K*-fold cross-validation procedure. Each observation was predicted by a model that was trained without using that observation in the training set. Consequently, OOF predictions provide an unbiased estimate of model performance on unseen data while utilizing all available observations for both training and validation across folds.

#### 4.4.2. Evaluation Metrics

Model performance was assessed using the coefficient of determination ($R^{2}$) and root mean squared error (RMSE).

### 4.5. Feature Ablation Test

To quantify the relative contribution of different feature groups to model performance, an ablation analysis was conducted using the trained ensemble model.

To evaluate the contribution of each feature category listed in Table 2, all variables belonging to one category were removed from the input set while the remaining variables were retained. The models were then retrained using the same cross-validation scheme. Feature importance was assessed by comparing the $R^{2}$ of the reduced model with that of the full model. The decrease in $R^{2}$ relative to the full model was used to quantify the contribution of each feature group to FW prediction performance.

### 4.6. Model Interpretation Using SHAP Values

To further interpret the contributions of individual features to FW prediction, SHAP analysis was applied to the trained ensemble models [37]. SHAP analysis was performed separately for the shoot FW and fruit FW estimation models. For each observation, the SHAP value represents the contribution of each feature to the deviation of the predicted value from the model baseline. SHAP plots were used to visualize the direction and magnitude of feature effects, allowing examination of how variations in morphological traits, temporal factor, and management factors influence FW predictions.

## 5. Conclusions

In this study, the FW estimation framework for sweet pepper was developed by integrating allometric modeling, machine learning, and data augmentation approaches. The ensemble model achieved the highest predictive accuracy, substantially outperforming traditional allometric models, particularly for fruit FW. Feature contribution analysis revealed that temporal progression was the dominant predictor of FW accumulation, while structural traits provided additional information for shoot FW estimation. Although the model was evaluated using data from a single greenhouse environment, the proposed framework provides a methodological approach for non-destructive FW prediction in greenhouse crops grown under diverse experimental treatments.

## Figures and Tables

**Table 1:** Experimental design and sample distribution by year, season, cultivar, treatment, and days after transplanting (DATs).

Year	Season	Cultivar	Treatment	DAT	Sample
2020	Spring	Scirocco	CT	30	3
				56	3
				84	3
				113	4
				133	20
			RB	84	3
				133	12
			FR	84	3
				133	12
2020	Winter	Mavera/Florate	CT	50	6
				80	6
				108	3
				153	15
			RB	108	3
				153	13
			FR	108	3
				153	12
2021	Spring	Mavera/Florate	CT	40	5
				60	5
				80	5
				100	5
				120	10
			RB	40	5
				60	5
				80	5
				100	5
				120	10
			FR	40	9
				60	10
				80	10
				100	10
				120	20
2021	Winter	Mavera	CT	150	9
			RB	150	9
			FR	150	36
2022	Spring	Mavera	CT	61	6

**Table 2:** Input and output factors used for machine learning model training.

Type	Category	Factor
Input	Basic traits	Plant height
		Basal stem diameter
	Structural traits	Main stem diameter
		Left stem diameter
		Right stem diameter
		Main stem length
		Left stem length
		Right stem length
	Temporal factor	Days after transplanting
	Management factors	Interlighting treatment
		Pruning status of main stem
Output	Target	Shoot fresh weight
		Fruit fresh weight

**Table 3:** Hyperparameters for Gaussian noise augmentation and variational autoencoder training. *n* and σ represent the number of samples in each group and the standard deviation, respectively.

Category	Parameter	Value
Gaussian noise augmentation	Minimum samples for noise augmentation	n≤10
	Noise standard deviation	0.05×σ
Variational Autoencoder	Minimum samples for training	n>10
	Latent dimension	8
	Hidden layer size	64, 32
	Epochs	500
	Batch size	32
	Learning rate	$1\times 10^{-3}$
	KL divergence weight (λ)	0.1

## Data Availability

The original contributions presented in this study are included in the article. Further inquiries can be directed to the corresponding author.
